# Supplementary material for: A systematic review to identify research priority setting in Black and minority ethnic health and evaluate their processes
Source: PLoS One. 2021 May 28;16(5):e0251685. doi: 10.1371/journal.pone.0251685 (PMC8162667; doi:10.1371/journal.pone.0251685)
Supplement: Attachment — Submitted filename: Minor_comments_SystematicReview_PONE-D-20-29158.docx [file pone.0251685.s002.docx]

| Page | Old | New |
| --- | --- | --- |
| 6 | setting partnership method) | setting partnership method). |
| 6 | this included studies where | , this included studies where |
| 15 | markers; audio recorders) | markers; audio recorders). |
| 16 | report any BAME PPI (20, 21, 25, 33, 35). | report any BAME PPI (20, 21, 25, 33, 35)~~.~~ |
| 20 | It should be noted that his issue is | It should be noted that this issue is |
| 20 | heard voices, Instead, | heard voices, instead, |
| 21 | this is a missed opportunity As well as | this is a missed opportunity. As well as |
| 22 | to generate research priorities. only | to generate research priorities, only |
| 23 | their involvement in the | their involvement in the ….. This |

Dear Author,

The systematic review “Research priority setting in Black, Asian and minority ethnic health” is a well-executed and written study. Research question, hypothesis, methods and results were clearly described. Hereby I am sending you my comments for a minor revision:

- Grammar errors
- *Since in the introduction the example of health conditions from UK Black African women was mentioned, the question rises why the search term “Africans” was not included in the search, to increase the reach of articles including this specific sub-group.*
- Introduction: “This systematic review is interested to see if there has been any progress in research priority process evaluation since then, with a specific focus on BAME health, given the stark increase in research priority setting in the past decade, along with an increase of discourse around evaluation of research priority setting initiatives” 🡪 *Recommendation to include the conclusion that since the publication of the narrative review from Bryant et al. (2014) and according to the results from this review (none of the studies fulfilled all the good practice criteria) barely any improvements have been made in terms of evaluation from prioritizations processes in exercises.*

*.*
